# KRASG12C mutant lung adenocarcinoma: unique biology, novel therapies and new challenges

**DOI:** 10.3389/pore.2023.1611580

**Published:** 2024-01-04

**Authors:** Judit Moldvay, József Tímár

**Affiliations:** ^1^ National Institute of Pulmonology, Budapest, Hungary; ^2^ Pulmonology Clinic, Szentgyörgyi A. University, Szeged, Hungary; ^3^ Department of Pathology, Forensic and Insurance Medicine, Semmelweis University, Budapest, Hungary

**Keywords:** KRAS, lung adenocarcinoma, G12C mutation, sotorasib, adagrasib

## Abstract

KRAS mutant lung cancer is the most prevalent molecular subclass of adenocarcinoma (LUAD), which is a heterogenous group depending on the mutation-type which affects not only the function of the oncogene but affects the biological behavior of the cancer as well. Furthermore, KRAS mutation affects radiation sensitivity but leads also to bevacizumab and bisphosphonate resistance as well. It was highly significant that allele specific irreversible inhibitors have been developed for the smoking associated G12C mutant KRAS (sotorasib and adagrasib). Based on trial data both sotorasib and adagrasib obtained conditional approval by FDA for the treatment of previously treated advanced LUAD. Similar to other target therapies, clinical administration of KRASG12C inhibitors (sotorasib and adagrasib) resulted in acquired resistance due to various genetic changes not only in KRAS but in other oncogenes as well. Recent clinical studies are aiming to increase the efficacy of G12C inhibitors by novel combination strategies.

## Introduction

**FIGURE 1:**
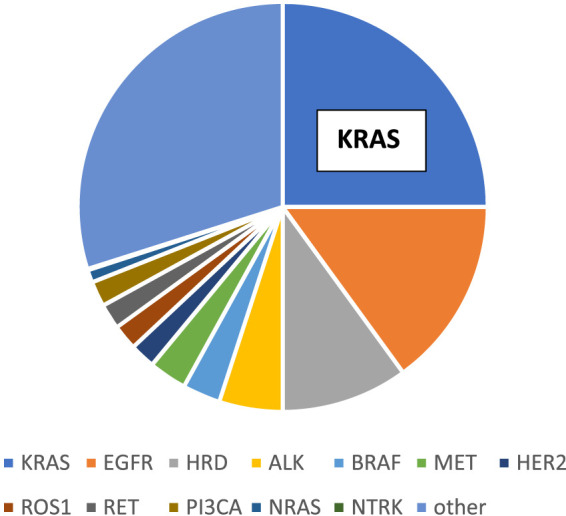
Molecular classification of lung adenocarcinoma.

The most frequent histological type of lung cancer is adenocarcinoma (LUAD) comprising half of the cases and the vast majority of the non-small cell lung cancers (NSCLC). The molecular classification of adenocarcinoma subgroup is established and is well known, where the most frequent genetic alteration among non-Asian patients is KRAS mutation (1/3) followed by EGFR (5%–15%) while in Asian patients EGFR mutation is the most frequent followed by KRAS mutation [1, 2]. Other relatively frequent mutations affecting BRAF and MET and by incidence followed by so called translocation cancers involving ALK/ROS1 less frequently RET or NTRK. At the same incidence levels, MET and HER2 amplifications also occur in this histological type [3]. It is of note that HRR mutations are also relatively frequent though less appreciated [4] (Figure 1). In the past decade target therapy changed the treatment of lung adenocarcinoma which left KRAS mutant lung cancer in an orphan status which changed recently significantly [5].

### Molecular epidemiology of KRASG12C mutant lung cancer

KRAS mutant lung cancer has three variants: type-1 is a characterized by mucinous histology with TTF1 expression, type-2 is characterized by high TMB and PDL1 expression while type-3 group contains KEAP mutation [6]. Other studies performed subclassification based on gene expression signatures and defined a p16 mutant, a p53 mutant and a STK11 mutant forms all having different expression profiles [7].

**FIGURE 2:**
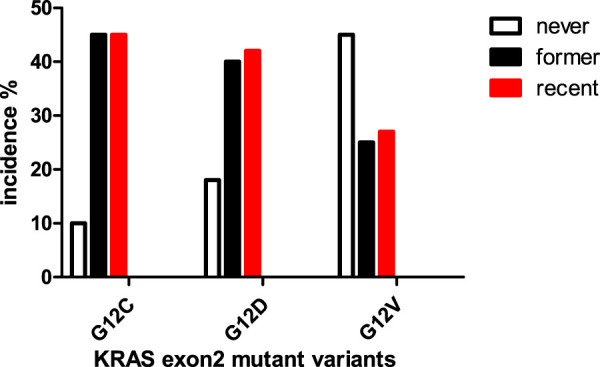
Connection between smoking history and KRAS mutant types in lung adenocarcinoma [10].

KRAS mutation in lung cancer has three predominant forms: the most frequent is G12C (∼40%) followed by ∼20–20%, G12D and G12V, respectively [1, 2, 8]. It is widely accepted that KRAS mutation in lung cancer is smoking associated but it is only proven for G12C while the G12D and G12V are associated with chromosomal instability and/or mismatch repair deficiency [9]. There is a clear association between smoking and allelic variants of mutant KRAS: among recent smokers far the most frequent is G12C mutation while among non-smokers G12V is the predominant (Figure 2). The presence of G12C mutation among non-smokers (∼10%) indicates the effect of passive smoking [10].

Various KRAS mutants are differ in biochemical and signaling functions: in G12C mutant the mitogenic RAS-RAF-MEK pathway is the most active, while in others the AKT signaling seems to be equally active, most probably due to changes in RAF affinity of the protein (Table 1, Ref [11]). Furthermore, individual mutants are characterized by differential alterations in GTP-ase activity or to sensitivity toward GAP proteins. Furthermore, the GDP/GTP exchange potential of the individual mutants seems also be different in various variants. There are other data supporting different lung carcinogenesis behind mutant KRAS variants: G12C mutation is associated with EGFR4 mutation, G12D mutations tend to have PDGRA mutation while G12V mutation containing tumor used to have PTEN mutation [1, 7]. Allelic imbalance of KRAS genes may also affect its function. In KRAS mutant lung adenocarcinoma heterozygous loss of the wild type allele is very frequent (∼75%) leaving the mutant allele the only functioning KRAS (a kind of homozygosity), whereas the copy gains of the mutant allele is much less frequent [12]. Other analyses defined the oncogenic driver roles of various KRAS mutant forms and found that G12C is a real major driver oncogene in lung cancer, unlike G12D/V which are only “mini-drivers,” cooperating with other mutant oncogens [13].

**TABLE 1:** Biochemical characteristics of KRAS mutant proteins [11].

KRAS	Wild type (G12)	G12C	G12D	G12V
GTP affinity	High	High	High	High
GDP/GTP exchange	Fast	Medium	Slow	Slow
GTP-ase activity	High	High	Decreased	Lost
GAP sensitivity	High	Lost	Lost	Lost
(B)RAF affinity	High	High	Decreased	Decreased

GAP, GTP-ase activating protein.

### Biology and therapeutic sensitivity of KRASG12C mutant lung cancer

Analysis of a large KRAS mutant LUAD database indicated that this type of lung cancer has increased potency to metastatize to the lung but decreased one to the liver and to invade the pleural surface [14]. Furthermore, it was shown that in case of bone metastases KRAS mutant status is an independent negative prognostic factor [14]. As far as the chemotherapeutic sensitivity concerns, most of the KRAS mutant variant containing tumors are equally sensitive toward platinum-based therapies, except the G12V mutant which seems to be more sensitive to this chemotherapy than others [10]. Another retrospective analysis tested the efficacy of bevacizumab in combination with chemotherapy and demonstrated that it is more efficient in KRAS wild-type tumors which was due to the resistance of the G12D mutant form [15]. Analysis of the treatment outcome of bone metastatic lung carcinoma patients indicated that the KRAS mutant tumors seems to be resistant to radiation therapy and to bisphosphonates [16] as it was predicted by the preclinical models [17]. A recent analysis of the G12D mutant lung cancers demonstrated that the density of CD8^+^ T cells, the TMB and the tumor cell expression level of PDL1 are lower as compared to other KRAS mutants including G12C [18]. More importantly, the efficacy of immune checkpoint inhibitors turned out to be poorer in G12D mutant lung cancers.

### Novel drugs to target mutant KRAS

#### The race for the G12C mutant KRAS inhibitor

**FIGURE 3:**
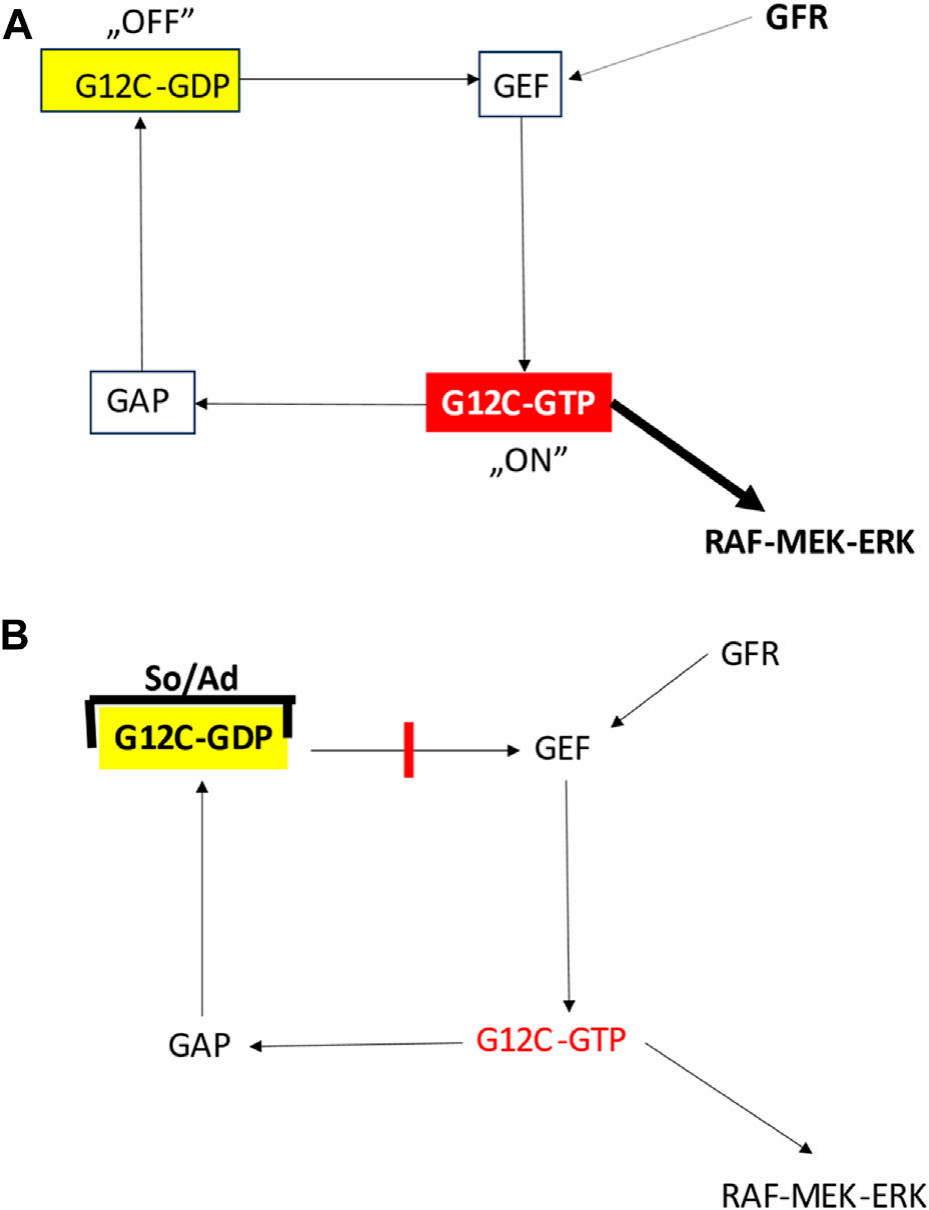
Effect of G12C inhibitors on the function of mutant KRAS protein [11]. **(A)** Function of the G12C mutated KRAS protein. **(B)** Function of the G12C mutated KRAS protein bound to inhibitors. Ad, adagrasib; GAP, GTP-ase activating protein; GEF, guanin nucleotide exchange factor; GFR, growth factor receptor; So, sotorasib.

Although it was considered undruggable, development of mutant KRAS inhibitors lastly became successful [19]. By the development of KRASG12C inhibitors. The challenge was here that—on the contrary to the various oncogenic tyrosine kinases where the increase kinase activity is the target—here in case of a GTP-ase the lost function is the target so a direct enzyme inhibitor is not an option. On the other hand, since the wild type KRAS is a critical signaling component of most of the normal cells, the inhibitor must be highly selective for the mutant isoform. As a result, a new class of inhibitors have been designed: the allele-specific (i.e., mutation specific) irreversible inhibitors. The idea was that since the KRAS is active in the GTP-bound state the novels drugs accumulate it in the off-state which is the GDP-bound KRAS (Figure 3).

The first in class of such KRASG12C inhibitor was published in 2013 [20] and a drug was approved for lung cancer in 2021 [20] which was a very rapid developmental process. The race was won by Amgen by a novel drug which is not only allele-specific (G12C) but also bind to a novel pocket (c95-99) critical in GTP-binding (ref [21], AMG-510, sotorasib). Preclinical data indicated that this novel inhibitor, not only blocks the mitogenic signaling (RAS-RAF-MEK) but is synergistic with platinum-based chemotherapy, with MEK inhibitors or with immune checkpoint inhibitors [21]. For the second place of this race arrived Mirati with a chemically distinct but functionally similar compound MRTX849/adagrasib which is characterized by very good pharmacological characteristics and which has a very good penetrance of the blood-brain barrier, forecasting its use for brain metastases [22]. It is of note that the half -life in the circulation of AMG510 is 5 h as compared to adagrasib’s 23 h. Meanwhile there are several other G12C inhibitors developed [23], some even reached clinical testing but only GDC-6036 exhibited early clinical efficacy [24].

#### Other mutant KRAS inhibitors

Developments in this filed continued by the G12D inhibitors which is far more frequent in other cancers but much less in lung adenocarcinoma. Unfortunately, irreversible inhibitors are nonexistent but a G12D selective inhibitor was developed: MRTX1133 which locks KRAS protein in the GTP-bound state which is in clinical development right now [25]. Furthermore, there are other novel inhibitors such as KRAS12D1-3 and RAS(ON)G12D [26].

#### Pan-RAS inhibitors

Other directions are the development of so-called pan-RAS inhibitors. BI-2852 induces homodimers of KRAS and turned out to be a KRASG12D selective inhibitor [27]. A real pan-KRAS inhibitor which even reached successful clinical testing is RMC-6236, a powerful RAS(ON) inhibitor which showed activity in G12V and other rare mutant forms [28].

#### Indirect RAS inhibitors

One of the main GTP-exchange protein of RAS is SOS1 and it serves as drug development target: there are several new molecules are on the market and some of them entered the clinic [29]. It would be interesting to see the side effect profile since these inhibitors are equally effective against all RAS isoforms and all variants, wild type or mutant. RAS proteins are phosphorylated at C32 of the exon2 by SRC and SHP2 phosphatase acting at this site. There are several SHP2 blockers in development and some of them entered clinical phase [30].

### Novel treatment options for KRASG12C mutant lung adenocarcinoma

In the past nearly 20 years, treatments targeting EGFR and ALK have already become part of everyday patient care, but at the same time, the use of targeted therapy against the driver mutation present in the largest proportion, the KRAS mutation, has only become a realistic possibility in recent years. We currently have the most experience with two KRAS inhibitors; these are sotorasib and adagrasib. The phase II trial for sotorasib was the Code-BreaK100, while that for adagarasib was the Krystal-1 clinical trial [31, 32].

The Code-BreaK100 trial investigated the activity of once-daily oral sotorasib 960 mg in patients with KRASG12C mutation-positive advanced NSCLC previously treated with platinum-based chemotherapy [31]. The primary endpoint was objective response (complete or partial) based on independent central review. Key secondary endpoints included duration of response, disease control (complete response, partial response, or stable disease), progression-free survival, overall survival, and patient safety. The predictive value of some biomarkers was also analyzed. Among the 126 enrolled patients, the majority (81.0%) had previously received platinum-based chemotherapy and PD-1 or PD-L1 inhibitors. According to the central review, 124 patients had measurable disease at baseline and the therapeutic response could be evaluated. An objective response was observed in 46 patients [37.1%; 95% confidence interval (CI), 28.6–46.2], including 4 (3.2%) complete responses and 42 (33.9%) partial responses shown. The median duration of therapeutic response was 11.1 months (95% CI, 6.9-not evaluable). Disease control occurred in 100 patients (80.6%; 95% CI, 72.6–87.2). Median progression-free survival was 6.8 months (95% CI, 5.1–8.2), and median overall survival was 12.5 months (95% CI, 10.0-not evaluable). Treatment-related adverse events occurred in 88 of 126 patients (69.8%), including a grade 3 event in 25 patients (19.8%) and a grade 4 event in 1 patient (0.8%). Therapeutic responses were also analyzed in subgroups defined by PD-L1 expression, tumor mutational burden (TMB), and concurrent STK11, KEAP1, or TP53 mutations. Based on all of this, in this phase II study, sotorasib therapy showed clinical benefit in patients with previously treated KRASG12C-mutated NSCLC without new patient safety signals [31].

The Krystal-1 study evaluated adagrasib (600 mg orally twice daily) in patients with KRASG12C-mutated NSCLC who had received prior platinum-based chemotherapy and anti-PD1 or anti-PD-L1 immunotherapy [32]. The primary endpoint was objective therapeutic response (ORR), assessed by an independent central review. Secondary endpoints included duration of response, progression-free survival, overall survival, and patient safety. A total of 116 patients with KRASG12C mutation-positive NSCLC were treated until October 15, 2021 (mean follow-up: 12.9 months); 98.3% had previously received both chemotherapy and immunotherapy. Of the 112 patients with measurable disease at baseline, 48 (42.9%) had a confirmed objective response with a median duration of 8.5 months [95% confidence interval (CI), 6.2–13.8], and the median progression-free survival was 6.5 months (95% CI, 4.7–8.4). As of January 15, 2022 (median follow-up, 15.6 months), the median overall survival was 12.6 months (95% CI, 9.2–19.2). In 33 patients with previously treated stable CNS metastases, the intracranial objective response rate was 33.3% (95% CI, 18.0–51.8). Treatment-related adverse events occurred in 97.4% of patients; grade 1 or 2 in 52.6%, grade 3 or higher in 44.8% (including two grade 5 events), and it became necessary to suspend medication in 6.9% of patients. Overall, in previously treated patients with KRASG12C-mutated NSCLC, adagrasib demonstrated clinical efficacy with no new patient safety alerts [32].

Below, we will review what differences can be verified between the two agents based on the results of these trials (Table 2). In phase II trials, the ORR was higher with adagrasib (43%) than with sotorasib (37%), and the rate of progressive disease (PD) was lower with adagrasib (16% for sotorasib vs. 5% for adagrasib), as shown in Table 2. However, in the absence of a head-to-head comparison, the results of such comparisons should be evaluated with caution [33]. Median PFS was similar between the two drugs (sotorasib, 6.6 months and adagrasib, 6.5 months). Drug-related adverse events were more common with adagrasib than with sotorasib, and, as a result, treatment interruption or dose reduction is more common with adagrasib (sotorasib, 22% and adagrasib, 52%). The confirmatory phase III trial for sotorasib was the Code-BreaK200 [34], while for adagrasib it was the Krystal-12 study.

**TABLE 2:** Summary of the clinical efficacies of sotorasib and adagrasib.

	Sotorasib	Adagrasib
	CodeBreaK100	KRYSTAL-1
N of patients	126	116
Primary endpoint	ORR	ORR
ORR (95% CI) (%)	37.1 (28.6–46.2)	43 (33.5–52.6)
DOR (95% CI) (month)	11.1 (6.9–NE)	8.5 (6.2–13.8)
DCR (95% CI) (%)	80.6 (72.6–87.2)	80 (70.8–86.5)
PFS (95% CI) (month)	6.6 (5.1–8.2)	6.5 (4.7–8.4)
OS (95% CI) (month)	12.5 (10.0–NE)	12.6 (9.2–19.2)
Follow-up (month)	15.3	12.9
Brain metastasis, n (%)	26 (20.6)	24 (21)
Intracranial ORR, DCR (%)	33, 85	12.5, 88
PD rate (%)	16.1	5
Dose reduction/suspension (%)	22.3	Reduction: 52; suspension: 61

ORR, objective response rate; DOR, duration of response; DCR, disease control rate; PFS, progression-free survival; OS, overall survival; CI, confidence interval; NE, not evaluated; PD, progressive disease.

In the Code-BreaK200 trial, between 4 June 2020 and 26 April 2021, 345 patients were randomized in a 1:1 ratio to the sotorasib (*n* = 171) or docetaxel (*n* = 174) arm. In the sotorasib group 169 (99%), and in the docetaxel group 151 (87%) patients received at least one course of treatment. After a median follow-up of 17.7 months, the study reached its primary endpoint, a statistically significant increase in PFS for sotorasib compared with docetaxel [median PFS 5.6 months (95% CI 4, 3–7.8) vs 4.5 months (3.0–5.7); HR: 0.66 (0.51–0.86) *p* = 0.0017]. Sotorasib was well tolerated, fewer grades 3 or worse [*n* = 56 (33%) vs *n* = 61 (40%)] and serious treatment-related adverse events compared with docetaxel [*n* = 18 (11%) vs *n* = 34 (23%)].

For sotorasib, the most common treatment-related adverse events of grade 3 or worse were diarrhoea [*n* = 20 (12%)], alanine aminotransferase increase [*n* = 13 (8%)], and aspartate aminotransferase increase [*n* = 9 (5%)]. For docetaxel, treatment-related adverse reactions of grade 3 or worse were neutropenia [*n* = 13 (9%)], fatigue [*n* = 9 (6%)], and febrile neutropenia [*n* = 8 (5%)]. In conclusion, sotorasib significantly increased progression-free survival and showed a more favorable safety profile compared to docetaxel in patients with advanced stage (IIIB/IV), good performance status (ECOG 0-1), KRASG12C-mutated LUAD who had already received platinum-based chemotherapy and immune checkpoint inhibitor therapy as first-line treatment and had no symptomatic brain metastases [34].

In the Krystal-12 trial, docetaxel was also the comparator agent and the inclusion criteria were the same as in the CodeBreak200 study, however, the randomization ratio was 2:1 in favor of adagrasib. Patients received 600 mg of adagrasib twice daily, and 75 mg/body surface area of docetaxel every 3 weeks. Adagrasib produced an ORR of 42.9% and a PFS of 6.5 months. Both drugs showed the already known side effect profile, the most common toxicities were diarrhea, musculoskeletal pain, fatigue and hepatotoxicity [35].

In NSCLC approximately 30%–40% of patients develop brain metastases during the course of the disease. In 2022, brain metastasis specific activity of adagrasib has been reported by Sabari et al. [36] Retrospectively, 374 NSCLC patients with KRAS mutations (149 with G12C mutation and 225 with non-G12C mutation) were analyzed for brain metastases. Overall, 40% of patients with KRASG12C or non-G12C mutations developed brain metastases during the follow-up period. 77% of patients had a diagnosis of synchronous brain metastases detected within 3 months of initial diagnosis. Brain metastasis occurred less frequently in NSCLC patients with KRAS mutations than in NSCLC patients with other oncogenic driver mutations [30]. In a retrospective review of 579 patients with metastatic NSCLC, the incidence of brain metastasis was highest in NSCLC patients with ROS1 (36%) and ALK (34%) mutations/fusions, followed by EGFR (28%) and KRAS (28%). In NSCLC without a driver oncogene, brain metastasis occurred in only 21% of patients [37]. The response of brain metastases to radiation therapy may vary depending on the driver oncogene. In an analysis by Arrieta et al., the response rate to radiotherapy was higher in NSCLC patients with EGFR (64.5%) or ALK (54.5%) mutations than in those without driver mutations (35%). However, in NSCLC patients with KRAS mutations, this rate is only 20%, which further emphasizes the need for effective treatments in this group [38].

Only limited data are available on the CNS activity of sotorasib in metastatic NSCLC. Although patients with active, untreated brain metastases were excluded from the Code-BreaK100 study, 2 of 16 patients with stable brain metastases had a complete response to therapy, and 12 achieved stable disease with sotorasib therapy, representing 88% of the patients with intracranial disease control [39]. In addition, several case studies have been published of patients with brain metastases in whom radiological regression was confirmed and symptoms resolved with sotorasib treatment [40, 41]. Yeh et al. reported a patient with NSCLC harboring a KRASG12C mutation with symptomatic leptomeningeal involvement and multiple brain metastases treated with sotorasib monotherapy [41]. The patient showed clinical improvement 2 weeks after the start of sotorasib treatment, and brain MRI showed clear radiological improvement in several metastatic foci and meningeal involvement. In this case, sotorasib was effective against untreated, symptomatic metastases. However, severe hepatotoxicity necessitated discontinuation of sotorasib, leading to disease progression. Therefore, although sotorasib is also effective in metastases affecting the central nervous system, further prospective studies are needed.

Negrao et al. studied the intracranial efficacy of adagrasib in KRASG12C-mutated NSCLC patients with untreated CNS metastases enrolled in the KRYSTAL-1 study [42]. 25 patients were enrolled and evaluated (mean follow-up, 13.7 months), and 19 patients had radiologically evaluable intracranial activity. Safety was consistent with previous reports for adagrasib: treatment-related grade 3 adverse events occurred in 10 patients (40%), grade 4 in 1 patient (4%), and there was no grade 5 adverse events. The most common CNS-specific adverse reactions were dysgeusia (24%) and dizziness (20%). Adagrasib showed an intracranial ORR of 42% and a DCR of 90%, as well as a PFS of 5.4 months and an OS of 11.4 months, which is promising for the treatment of patients with untreated CNS metastases.

The clinical trial results of the KRAS inhibitors sotorasib and adagrasib are promising, however, currently they are inferior to EGFR inhibitors or ALK inhibitors in terms of both therapeutic duration (PFS, OS) and side effect profile. Further extensive studies—mainly targeting predictive markers and resistance mechanisms—are necessary in order to be able to treat permanently and effectively this large group of patients with a good quality of life.

### Primary and acquired resistance mechanisms

#### Primary resistance

There are characteristic co-occurring mutations in KRAS mutant lung cancer such as STK11 and KEAP1. STK11 mutation was shown to be associated with resistance to immunotherapy [43]. In the CodeBreak100 study the association of STK11 and KEAP1 mutations have been evaluated in relation to the efficacy of sotorasib and found that the lowest response rate was found in tumors having KEAP1 mutation/STK11 wild type genotype while the highest was seen in tumors with STK11mutant/KEAP1 wild type genotype [44]. A recent genomic analysis of a large G12C mutant lung cancer cohort treated with G12C inhibitors revealed that co-occurring mutations of KEAP1, SMARC4 and CDKN2A were independent negative predictive factors of inhibitor efficacy while mutations in the DDR genes were positive predictive ones [45].

#### Acquired resistance

Acquired resistance to sotorasib treatment of lung cancer patients had various pathomechanisms At the first place it was found the disappearance of G12C mutation from cancer cells or the amplification of the wild type KRAS gene. Other KRAS-related genetic alterations were the acquired novel mutation types (G13V, G12D, G12V, V8L, V141I) or the novel mutations affecting NRAS. Furthermore, mutations of the EGFR signaling pathway members such as EGFR or BRAF are also occurred [46]. Although at not high frequency, but amplifications of MET or HER2 have also been reported [47, 48].

Upon adagrasib resistance it was described histological transformation from adenocarcinoma to squamous [49] a bit similar to what was seen in case of EGFR inhibitor resistance. It can occur most probably in those cases where the original tumor is a combined adenosquamous variant since KRAS mutation is adenocarcinoma specific genetic alterations. In case of acquired resistance to adagrasib at first place also novel KRAS mutations have been identified (G12D/R/W, G13D, Q61H, R68S, H95D/Q/R, Y96C). The resistance mechanism does not involve the EGFR signaling instead the RET signaling with mutations affecting RET, BRAF and MAP2K1. Furthermore, gene amplification here also involved MET but interestingly there were several gene fusions in the resistant tumors involving, ALK, RET, FGFR3 and BRAF [49].

The resistance mutations of KRAS can be classified into three main categories. Mutations in the codon12 or codon61 decrease the potential of the KRAS protein to hydrolyze GTP. Mutations at codon 13 increase the GDP-GTP exchange, while mutations at R68, H95, Y96 and Q99 decreases the affinity of the inhibitors.

It is interesting that various mutational profiles of the KRAS mutant lung cancers affect the development of resistance to sotorasib or adagrasib [49] The H95 mutations may confer resistance to adagrasib but does not affect the activity of sotorasib. On the other hand, G13D, R68M, A59S/T mutations confer sotorasib resistance but retain adagrasib sensitivity [48]. Finally, m72 or Q99 mutations cause adagrasib resistance but do not affect sotorasib sensitivity [50]. Based on these data it can be hypothesized that the development of acquired resistance could be treated by sequential use of the other G12C inhibitor.

### Developing combinational approaches

The observed clinical efficacy and the developing resistances both stimulated novel clinical approaches to improve the efficacy of G12C inhibitors sotorasib and adagrasib (Table 3) [51]. Since G12C mutant lung cancer is an immunologically hot tumor it was evident to start combinations with PD1/PDL1 inhibitors: in case of sotorasib the combination partner is AKG404 (a PD1 inhibitor) in case of adagrasib the partner is Pembrolizumab (also a PD1 inhibitor). Since one of the resistance mechanisms of G12C inhibitors involves the reactivation of EGFR signaling pathway, sotorasib is now clinically tested in combination with afatinib (an EGFR tirozin kinase inhibitor). In case of both G12C inhibitors the efficacy against colorectal cancer is a significant problem therefore combinational trials using anti-EGFR antibodies. Other interesting novel combination involves bevacizumab (anti-VEGF) since this therapy was shown to be inactive in KRAS mutant lung cancer [15]. Furthermore, combinational trials of G12C inhibitors are already initiated with traditional chemotherapies such as carboplatin/pemetrexed. Since acquired resistance to G12C inhibitors may involve reactivation of alternative signaling pathways such as PI3KCA (sotorasib) combination with mTOR inhibitor seems to be a rational approach. It is a completely different approach to increase the KRAS inhibitory efficacy of G12C inhibitors by either SOS1 inhibitors (to block GEF protein activation) or with SHP2 inhibitors (to block reactivation mechanisms) [51]. Since these approaches are pan-RAS targeted, it will be an interesting issue to see that for the prize of increased G12C inhibition what kind of prize can be paid in terms of side effects.

**TABLE 3:** Clinical developments of G12C inhibitor combinations [51].

G12C inhibitor	Partner	Function	NCT	Clinical phase	Cancer
sotorasib	AMG404	PD-1 inhibitor	03600883	I/II	NSCLC
	carboplatin/pemetrexed	chemotherapy	(Japan)	II	NSCLC
	palbociclib	CDK4/6 inhibitor	05178888	I/Ib	solid tumor
	afatinib	EGFR-inhibitor	04185883	Ib/II	NSCLC
	panitumumab	anti-EGFR	05198934	III	CRC
	everolimus	mTOR inhibitor	04185883	Ib/II	solid tumor
	RMC-4630	SHP2 inhibitor	04185883	Ib/II	solid tumor
	bevacizumab	anti-VEGF	05180422	I/II	NSCLC
adagrasib	pembrolizumab	anti-PD1	046113596	II	NSCLC
	cetuximab	anti-EGFR	04793958	III	CRC
	TNO-155	SHP2 inhibitor	04330664	I/II	solid tumor
	BI-17011963	SOS1 inhibitor	04975256	I/Ib	solid tumor

CRC, colorectal cancer; NSCLC, non-small cell lung cancer.

## Conclusion

KRAS mutant lung adenocarcinoma is the most frequent molecular subtype of lung cancer but it is still a heterogenous entity since the individual allelic variants are biologically heterogenous. The most frequent allelic variant of KRAS mutant lung cancer is the smoking related G12C which became the focus of the development of mutant-specific irreversible KRAS inhibitors. More importantly, two of the G12C inhibitors, sotorasib and adagrasib were effective clinically in advanced G12C mutant lung adenocarcinoma patients resulting in conditional approval (linked to annual reporting of the expected clinical efficacy). Meanwhile, similar to other target therapies, upon administration of G12C inhibitors clinical resistance develops which is due to various biological processes predominated by secondary mutations of the KRAS gene. Since the clinical efficacy of G12C inhibitors is not overwhelming, there is a room for improvement which is the bases of development of various combination approaches of G12C inhibitors including immunotherapeutic agents, EGFR inhibitors or RAS signaling modulators. Since mutant KRAS was long considered undruggable, the development and the clinical success of G12C inhibitors pave the way for the development of non-G12C mutant KRAS inhibitors, opening the door for a new era of target therapies aiming at the most frequently mutated human oncogene in various cancers including the lung adenocarcinoma.

## Abbreviations

CI, confidence interval; CNS, central nervous system; DCR, disease control rate; GFR, growth factor receptor; HR, hazard ratio; HRD, homologous recombination repair deficiency; HRR, homologous recombination repair; LUAD, lung adenocarcinoma; NSCLC, non-small cell lung cancer; ORR, overall response rate; OS, overall survival; PD, progressive disease; PFS, progression-free survival; TMB, tumor mutational burden.
